# Genetic Variability in NKG2 Receptors and Their Ligands: Associations with SARS-CoV-2 Infection and COVID-19 Severity

**DOI:** 10.3390/genes16101193

**Published:** 2025-10-13

**Authors:** Jagoda Siemaszko, Katarzyna Grad, Jerzy Świerkot, Katarzyna Bogunia-Kubik

**Affiliations:** 1 Laboratory of Clinical Immunogenetics and Pharmacogenetics, Hirszfeld Institute of Immunology and Experimental Therapy, Polish Academy of Sciences, 53-114 Wroclaw, Poland; jagoda.siemaszko@hirszfeld.pl (J.S.);; 2 Department of Rheumatology and Internal Medicine, Wroclaw Medical University, 50-556 Wroclaw, Poland

**Keywords:** NK cells, NK cell receptors, SARS-CoV-2, COVID-19, HLA-E, NKG2C, viral infection

## Abstract

Background: The emergence of the COVID-19 pandemic has accelerated research into diverse immune response mechanisms. One key area of interest is the regulation of cytotoxic activity by Natural Killer (NK) cells. These cells rely on a dynamic interplay between activating and inhibitory surface receptors that recognize specific ligands on target cells. Among these, receptors from the NKG2 family are particularly important, as maintaining their proper balance and function is essential for controlling NK cell cytotoxicity. Methods: In this study we employed qPCR to assess the genetic variability using single-nucleotide polymorphisms (SNPs) of NKG2A and NKG2D receptors and their ligands HLA-E and MICA/MICB. NKG2C deletion was determined by PCR-SSP, and serum-soluble levels of HLA-E and MICA/MICB molecules were measured by ELISA and Luminex methods. Results: Genotyping studies revealed that both *NKG2A* rs7301582 *T* and *HLA-E* rs1264457 *A* (HLA-E*01:01) alleles were predominant among infected individuals (OR = 2.21, *p* = 0.0258 and OR = 2.84, *p* = 0.0257, respectively). In contrast to *MICB* rs1065075 *A*, the *MICA* rs1051792 *A* (129Met) allele was most commonly found in hospitalized patients (OR = 14.95, *p* = 0.0114). The presence of the *NKG2C del* variant tended to be associated with an increased risk of SARS-CoV-2 infection (OR = 2.02, *p* = 0.0694). Moreover, higher concentrations of serum-soluble MICB was detected in infected individuals as compared to the control group (*p* = 0.008). Conclusions: Genetic variability of NK cell receptors and ligands as well as serum levels of their soluble forms showed associations with the risk of development of COVID-19 and the severity of its symptoms.

## 1. Introduction

One of the main effector lymphocytes are NK cells serving as natural sentinels of immunity. NK cells constitute a critical component of the innate immune system, playing a key role in the defence against viral infections. They recognize and eliminate virus-infected cells through direct cytotoxicity, mediated by perforin and granzymes, as well as through antibody-dependent cellular cytotoxicity (ADCC) [1]. Additionally, NK cells produce cytokines such as interferon-gamma (IFN-γ), which enhance antiviral immunity by activating other immune cells [2]. Their rapid response is particularly important in early infection stages before the adaptive immune system fully engages. However, some viruses, like cytomegalovirus (CMV) and human immunodeficiency virus (HIV), have evolved mechanisms to evade NK cell-mediated immunity, highlighting the complexity of their role in viral defence [3]. NK cell activity is tightly regulated by a balance of activating and inhibitory receptors, which detect stress signals or the absence of major histocompatibility complex (MHC) class I molecules on infected cells. These cell receptors are categorized into several major groups, each playing a distinct role in immune regulation [4]. Killer cell immunoglobulin-like receptors (KIRs) are a diverse family of inhibitory and activating receptors that recognize MHC class I molecules, helping NK cells differentiate between healthy and abnormal cells [5]. Natural Cytotoxicity Receptors (NCRs), including NKp30, NKp44, and NKp46, are potent activating receptors that detect stress-induced ligands on virus-infected and tumour cells, triggering NK cell-mediated cytotoxicity [6]. Immunoglobulin-like transcripts (ILTs), such as ILT2 and ILT4, primarily function as inhibitory receptors by interacting with classical and non-classical MHC molecules, contributing to immune tolerance [7,8]. NKG2 receptors, including NKG2A, NKG2C, and NKG2D, are C-type lectin-like receptors that bind to HLA-E or stress-induced ligands like MICA/MICB, modulating NK cell activation in response to infection or malignancy. The interplay between these receptor groups ensures a fine balance between immune surveillance and self-tolerance [9].

NKG2A is an inhibitory receptor that forms a heterodimer with CD94 and binds to HLA-E, a non-classical MHC class I molecule. When engaged, it suppresses NK cell activity, preventing unnecessary immune responses and maintaining tolerance to healthy cells [10,11,12]. NKG2C, also paired with CD94, is an activating receptor recognizing the HLA-E molecule as well. Its activation enhances NK cell cytotoxicity and cytokine production, particularly in the context of viral infections, such as CMV, which is known to drive the expansion of NKG2C+ NK cell subsets. Leader peptides, mainly derived from HLA class I signal sequences, are generated during protein translocation. The hydrophilic fragment is further processed by the proteasome, producing leader peptides. These are then transported to the endoplasmic reticulum, where they bind HLA-E molecules, enabling their surface expression [13]. NKG2D is a highly potent activating receptor that binds to stress-induced ligands, such as MICA, MICB, and ULBPs, which are upregulated on virus-infected and tumour cells. This receptor plays a crucial role in immune surveillance by promoting NK cell-mediated cytotoxicity and immune responses against malignancies and infected cells [9].

The coronavirus disease 2019 (COVID-19) pandemic has highlighted the critical role of NK cell receptors in immune responses to viral infections. NKG2A, an inhibitory receptor, has been shown to be upregulated in severe COVID-19 cases, contributing to NK cell exhaustion and impaired viral clearance [14]. In contrast, NKG2C, an activating receptor, is often expanded in individuals with prior CMV infections and has been associated with a more robust NK cell response against SARS-CoV-2 [15]. The MICA/MICB-recognizing NKG2D receptor promotes cytotoxicity and cytokine production; however, SARS-CoV-2, like other viruses, may evade immune detection by downregulating NKG2D ligands, weakening the NK cell-mediated defence [16]. Understanding the interplay between NKG2 receptors and viral infections could provide insights into disease severity and potential immunotherapeutic strategies for COVID-19 and other emerging pathogens [17].

Here, we analyze the possible associations between the genetic variability of NKG2 receptors as well as their ligands and the incidence and severity of SARS-CoV-2 infection in a Polish cohort. In addition, the soluble forms of HLA-E and MICA/MICB are studied.

## 2. Materials and Methods

### 2.1. Individuals Studied

The study group consisted of representatives of the medical staff from Wroclaw Medical University with an average age of 47 years. Eligibility criteria for this study were as follows: age > 18 y/o, active employment in the hospital, and signature consent to the study. A total of 192 individuals divided into four groups participated in this study. Three groups were selected based on the severity of COVID-19 symptoms: (i) asymptomatic (37 cases; females (F)/males (M) 12/25); (ii) those who received home treatment (70 patients; F/M 23/47); (iii) hospitalized (10 patients; F/M 0/10). The fourth (iv) group constituted 75 healthy controls (non-infected individuals; F/M 25/50). Classification into study groups was performed by qualified, professional clinicians.

The following information was obtained regarding the course of SARS-CoV-2 infection: method confirming SARS-CoV-2 infection (positive PCR test result based on a nasopharyngeal swab, positive antigen test result from a nasopharyngeal swab, or positive test result for antibodies against SARS-CoV-2), symptoms of SARS-CoV-2 infection, and severity of the course of the disease. A participant with a history of COVID-19 infection was defined as a person who (1) had a positive PCR test and/or antigen test based on a nasopharyngeal swab and/or (2) had a positive test for immunoglobulin G (IgG) and/or IgM antibodies against SARS-CoV-2 at the time of this study. Other participants who did not meet the above conditions were considered as those without a history of COVID-19 infection.

This study received approval from the institutional ethics committee of Wroclaw Medical University, Wroclaw, Poland (approval No. KB 634/2020 and approval No. KB 157/2023), and adhered to the ethical guidelines of the Declaration of Helsinki.

### 2.2. DNA Isolation

For genetic variability studies, genomic DNA isolated from whole blood collected on ethylenediaminetetraacetic acid (EDTA) tubes was used. The column method was applied for DNA isolation (NucleoSpin Blood kit, MACHEREY-NAGEL, Dueren, Germany), following the manufacturer’s instructions. The purity of isolated DNA and its concentration were assessed using a DeNovix spectrophotometer (DeNovix, Wilmington, DE, USA).

### 2.3. SNP Genotyping

Four SNPs within genes coding for NK cell receptors and their ligands were chosen. SNP selection was performed using online databases, prediction tools (SNPinfo Web Server [18]), the literature overview, and the results of our previous studies. Genotyping was performed using LightSNiP assays (TibMOLBIOL, Berlin, Germany) on a LightCycler480 II Real-Time PCR system (Roche Diagnostics, Rotkreuz, Switzerland). Detailed characteristics of selected SNPs are shown below (Table 1). Negative control was included in all experiments.

### 2.4. NKG2C Deletion Detection

Detection of *NKG2C* deletion was performed using PCR-SSPs (Polymerase Chain Reaction with Sequence Specific Primers) with two pairs of oligonucleotides as previously described [19,20]. Primers specific for *NKG2C* deletion were (i) KLRdelF 5′-ACTCGGATTTCTATTTGATGC-3′ and (ii) KLRdelR 5′-ACAAGTGATGTATAAGAAAAAG-3′, while for the wild type they were (i) KLRFg669 5′-CAGTGTGGATCTTCAATG-3′ and (ii) KLRR+ 135 5′-TTTAGTAATTGTGTGCATCCTA-3′. PCR products were electrophoresed in 2% agarose gels with 1× TBE buffer stained with SimplySafe™ (EURx, Gdańsk, Poland) and visualized by UV exposure.

### 2.5. Soluble Forms of HLA-E and MICA/MICB Ligands

Serum samples were collected for analyzing concentrations of soluble forms of HLA-E and MICA/MICB ligands and stored at −80 °C before use. For measurements of the serum concentration of soluble HLA-E, the commercially available enzyme-linked immunosorbent assay (ELISA) method was adopted (ELK Biotechnology, Sugar Land, TX, USA) on 40 representative samples. The experiment was performed following the manufacturer’s protocol. Absorbance was measured at λ = 450 nm using a Sunrise microplate reader (Tecan, Männedorf, Switzerland). The same 40 serum samples were incorporated for measurements of MICA and MICB levels using the Luminex^®^ Discovery Assay (bio-techne^®^, R&D SYSTEMS, Minneapolis, MN, USA). Samples were prepared in a 2-fold dilution and measured using a Luminex 200 instrument (Luminex Corp., Austin, TX, USA). Median fluorescence intensity (MFI) was calculated using the xPonent v.4.2 software. All measurements were made in duplicate.

### 2.6. Statistical Analysis

Genotype and allele frequencies of studied polymorphisms were calculated using Fisher’s exact test. For the results of sHLA-E, sMICA, and sMICB concentrations, the nonparametric Mann–Whitney test for continuous variables was used. Programmes used for calculations and data visualizations were RStudio v.4.2 and GraphPad Prism v.9.0. A *p*-value at <0.05 was considered statistically significant.

## 3. Results

### 3.1. SNP Genotyping

**Figure 1:**
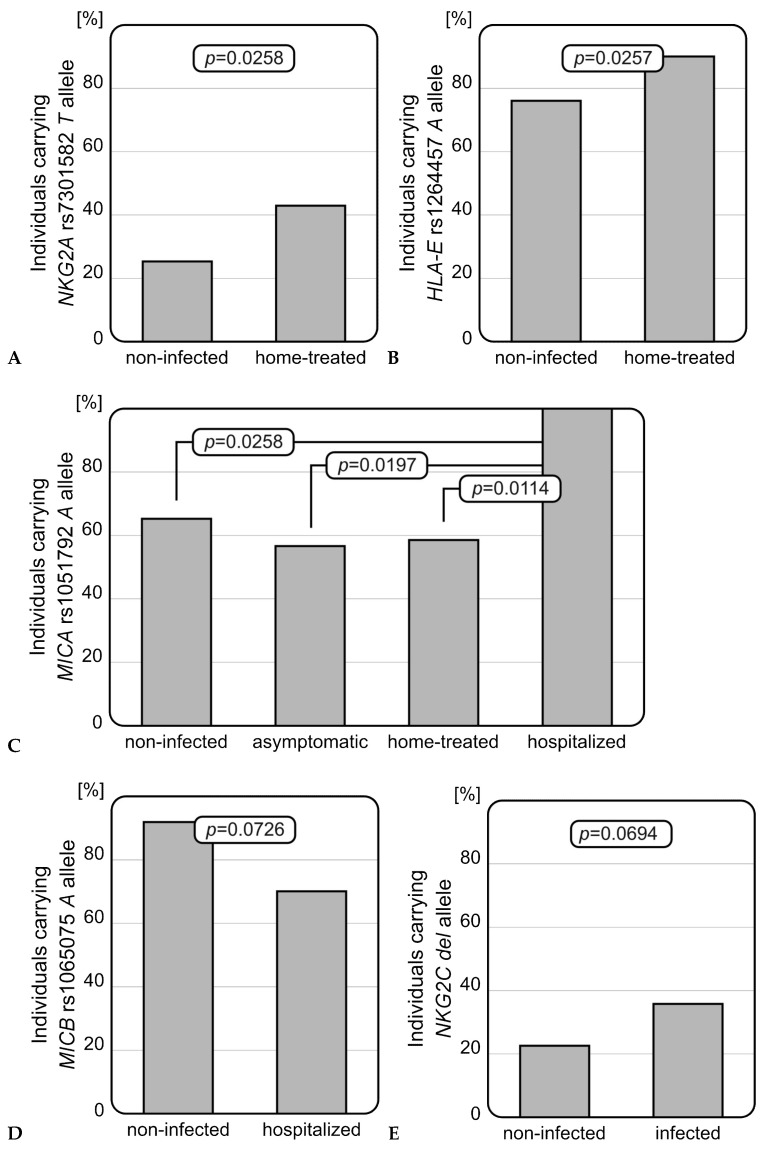
Significant relationships observed for selected polymorphisms. (**A**) *NKG2A* rs7301582 polymorphism in COVID-19 severity: the rs7301582 *T* allele was more frequently detected among infected individuals who received home treatment than in non-infected subjects. (**B**) *HLA-E* rs1264457 polymorphism in COVID-19 severity: rs1264457 *A* allele was more frequently present among home-treated patients than healthy individuals. (**C**) *MICA* rs1051792 polymorphism in COVID-19 severity: rs1051792 *A* allele was most common among hospitalized patients, when compared with non-infected individuals or patients with mild infection. (**D**) *MICB* rs1065075 polymorphism in COVID-19 severity: rs1065075 *A* allele was less frequently detected among hospitalized patients. (**E**) Difference in *NKG2C* deletion frequency between non-infected and infected individuals: patients with diagnosed SARS-CoV-2 infection carried the *NKG2C del* variant more frequently.

Genotyping studies revealed that infected individuals who received home treatment carried the *NKG2A* rs7301582 *T* allele more frequently than those without SARS-CoV-2 infection (OR = 2.21, *p* = 0.0258, Figure 1A). This genetic variant was present in 42.9% (30/70) of home-treated patients, while among the healthy individuals, it was detected in 25.3% (19/75). Similarly, patients who received home treatment were characterized with an increased frequency of the *HLA-E* rs1264457 *A* allele when compared to healthy controls (OR = 2.84, *p* = 0.0257, Figure 1B). Within the controls, *HLA-E* rs1264457 *A* and *G* alleles were uniformly distributed. The *MICA* rs1051792 SNP analysis showed that among infected individuals who were hospitalized, the *A* allele occurred with the highest frequency of all studied groups—all hospitalized patients carried at least one *MICA* rs1051792 *A* allele (OR = 14.95, *p* = 0.0114, Figure 1C). In contrast to the *MICA* rs1051792 *A* variant, the *MICB* rs1065075 *A* allele seemed to play a protective role and tended to be more frequent among non-infected individuals as compared to those who required hospitalization (OR = 0.21, *p* = 0.0726, Figure 1D), suggesting a more unfavourable role of the *MICB* rs1065075 *GG* homozygosity. Analyses of the genetic distributions of selected SNPs did not show any other differences between a genetic variant and disease severity. Additionally, an interesting association was observed within the control group. The *A* allele of *MICA* rs1051792 SNP prevailed among women when compared to the men (OR = 3.083, *p* = 0.0389). Such a relationship was not observed for any other of the analyzed SNPs. No associations for the genetic distribution of *NKG2D* rs1049174 SNP were found. Detailed results of the SNPs genotyping are shown below (Table 2).

### 3.2. NKG2C Deletion

Patients infected with SARS-CoV-2 (independently of the severity of the infection) were characterized by a more frequent presence of the *NKG2C* deletion than healthy individuals. Although this finding did not reach statistical significance, a strong tendency was observed (OR = 2.02, *p* = 0.0694, Figure 1E). The *NKG2C del* variant occurred with nearly two times higher frequency among infected patients than non-infected individuals (37.2% vs. 22.7%).

### 3.3. Serum sHLA-E, sMICA, and sMICB Concentrations

**Figure 2:**
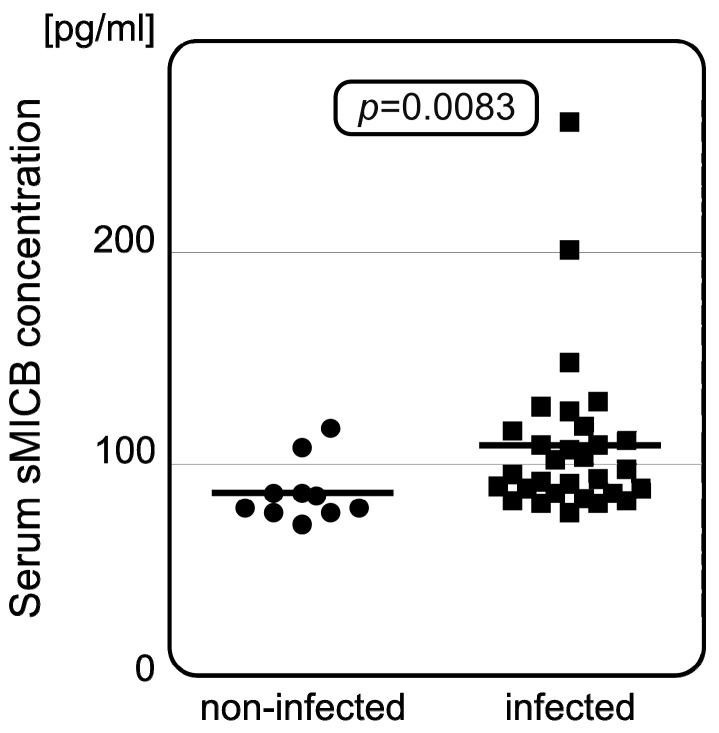
Difference in serum sMICB concentrations between non-infected and infected patients. Infected individuals have increased serum sMICB levels when compared to those without infection. The dots and squares show individual measurements with horizontal lines as median values for each group.

Serum concentrations of sHLA-E, sMICA, and sMICB were measured in 40 representative samples (including controls). Detailed results of the measurements are shown below (Table 3). We did not observe any association between serum sHLA-E levels and patients’ *HLA-E* genotype or COVID-19 severity. Furthermore, serum sHLA-E concentrations did not differ between infected individuals and controls. Serum sMICA also does not seem to affect the risk of infection. However, we observed that SARS-CoV-2-infected individuals were characterized with significantly higher serum sMICB concentrations than healthy controls (*p* = 0.0083, Figure 2), but, similarly to sHLA-E and sMICA, no relationship was found between *MICB* rs1065075 SNP and sMICB levels.

## 4. Discussion

NK cells constitute a critical component of the innate immune system, playing a key role in the defence against viruses, including SARS-CoV-2. Among SARS-CoV-2-infected patients, NK cells show a rather mature profile and are characterized with activating potential, although infected individuals have a lower number of peripheral NK cells than healthy ones, and the frequency of NK cells was associated with disease severity [21,22,23,24]. It has also been reported that IFN-γ and perforin release rates were significantly lower in patients with SARS-CoV-2 earlier during infection, which indicates insufficient clearance of virus-infected cells by the NK cells [25].

The present work aimed to assess the effect of genetic variability in genes coding for NKG2 receptors (NKG2A, NKG2C, and NKG2D), as well as their ligands (HLA-E, MICA, MICB), and the serum concentrations of these ligands on the incidence of SARS-CoV-2 infection and severity of COVID-19 in representatives of the Polish population. We found that *NKG2A* rs7301582 *T*, *HLA-E* rs1264457 *A*, *MICA* rs1051792 *A*, and *NKG2C del* genetic variants were more commonly present among SARS-CoV-2-infected individuals. In comparison with individuals without a SARS-CoV-2 infection, home-treated patients were characterized by an increased frequency of the *NKG2A* rs7301582 *T* allele (*p* = 0.0258) and *HLA-E* rs1264457 *A* allele (*p* = 0.0257). Furthermore, we found that all hospitalized individuals carried at least one *MICA* rs1051792 *A* allele and, within this group, this variant occurred with the highest frequency (*p* = 0.0114). As for *MICB*, the rs1065075 SNP could potentially be linked to COVID-19 severity. The presence of the rs1065075 *A* allele was more frequently observed among non-infected individuals when compared to hospitalized patients. Hospitalized patients, on the other hand, carried the rs1065075 *GG* genotype more commonly (*p* = 0.0726). The *NKG2C del* variant occurred with higher frequency among infected individuals, although this observation did not reach statistical significance (*p* = 0.0694). Results obtained within this study indicate that the presence of deletion within the *NKG2C*/*KLRC2* gene, as well as *NKG2A* rs7301582, *HLA-E* rs1264457, *MICB* rs065075, and *MICA* rs1051792 SNPs, could be associated with COVID-19 susceptibility and the severity of the disease. However, on the other hand, in our Polish cohort, no significant association was detected with functional rs1049174 Val129Met polymorphism within the *NKG2D*/*KLRK2* gene coding for another activating receptor of this NKG2 family.

Recently, the presence of *NKG2C* deletion was indicated as a risk factor for COVID-19 by Vietzen et al. [15]. Supporting this finding, we also observed an association between the frequency of the NKG2C *del* variant and SARS-CoV-2 infection development. Infected individuals carried the *del* variant almost two times more frequently than those without infection. It was reported that deletion within the *NKG2C*/*KLRC2* gene results in an impaired receptor structure and its decreased expression [26]. In effect, this mutation lowers the NK cells’ affinity to the HLA-E molecule through the NKG2C receptor. Thus, it appears that the presence of a fully expressed activating NKG2C receptor seems to be important for curing the infection.

As for inhibitory NKG2A, a representative of NKG2 receptors, our previous study, performed on pediatric hematopoietic stem cell transplantation (HSCT) recipients, showed that carriers of the *NKG2A* rs7301582 *T* allele were more prone to severe acute graft-versus-host disease development, suggesting an unfavourable impact of this genetic variant [27]. Here, we also showed a negative role of this *NKG2A* rs7301582 *T* allele, as it was more frequently present among infected individuals (who received home treatment) than those without SARS-CoV-2 infection (*p* = 0.0258). Although studies on rs7301582 polymorphism are still limited, the other *NKG2A* SNP, rs2734440, showed protective associations in SARS-CoV-2 infection, as the *G* allele was more frequently detected in non-infected individuals [28].

Interestingly, a similar association was observed for the gene encoding the HLA-E molecule, which is also the ligand of this NKG2A receptor. In the present study, the *HLA-E* rs1264457 *A* (*01:01) allele was more frequently found among home-treated SARS-CoV-2-infected individuals than in healthy controls (*p* = 0.0257). Vietzen et al. showed that the frequency of HLA-E*01:01 was increased in hospitalized COVID-19 patients [15]. Thus, both studies seem to present a rather unfavourable impact of the HLA-E*01:01 and document its association with more severe SARS-CoV-2 infection. The negative effect of the HLA-E*01:01 allele on viral infection was also observed in our previous analyses, where this genetic variant was correlated with an increased risk of CMV reactivation after allogeneic HSCT [20].

Moreover, healthy controls of the present study were characterized by uniform distribution of both analyzed HLA-E alleles, which stands in line with the general population frequency of *HLA-E* rs1264457 variants. HLA-E *A*/*01:01 and *G*/*01:03 alleles differ by the exchange of amino acids at position 107, causing an arginine (HLA-E*01:01) to glycine (HLA-E*01:03) substitution. Interestingly, it was observed that the *HLA-E* rs1264457 *G*/*01:03 allele is characterized by higher surface expression and affinity to peptide [13]. The presence of this genetic variant—HLA-E*01:03 allele—shows a rather protective effect, as was observed also in an allogeneic transplant setting in the context of acute graft-versus-host disease [29] or in HIV infection [30]. HLA-E surface expression depends on the availability of suitable binding peptides. By presenting intracellular peptides, HLA-E signals the status of cellular protein synthesis, particularly the maturation of HLA class I molecules [13]. HLA-B is characterized by a specific methionine (M)/threonine (T) dimorphism at position -21 (with the M variant connected with functional binding to HLA-E and the T variant with non-effective binding). Indeed, we observed that this HLA-B -21 dimorphism leads to differences in IFN-γ release as well as TNF-α concentrations, suggesting its impact on the cellular response in kidney transplant recipients after COVID-19 vaccination [31]. A m recent study performed on hospitalized SARS-CoV-2 infection patients does not, however, support this observation [32].

Although we have not observed any significant effect of the *NKG2D* rs1049174 SNP on COVID-19 manifestation, a study by Tripathy et al. showed that *NKG2D* rs7980470 intronic polymorphism affects the risk of SARS-CoV-2 infection, indicating the potential role of this activating receptor [28]. Indeed, polymorphisms within *NKG2D* were found to be associated with various infections, including CMV [33], hepatitis B [34], or HPV-related cancers [35], although we did not observe any association between the studied *NKG2D* variants and COVID-19. Some relationships were, however, observed, when their ligands were considered. The results of *MICA* rs1051792 *SNP* analysis revealed that the *A*/Met allele can be associated with COVID-19 severity, as was detected in all hospitalized patients. A negative effect of the *MICA* rs1051792 *A* allele was also observed in the context of autoimmune disease. We note that the presence of the *MICA A*/Met allele may also have some functional implications and leads to a stronger interaction with the NKG2D receptor, resulting in increased activating potential of this receptor and, therefore, higher NK cell activity [36]. It also supports NK cell degranulation and production of IFN-γ. MICA and MICB variants were previously associated with SARS-CoV-2 infection, especially with symptomatic infections [37,38]. Additionally, some polymorphic sites, including the Met129 variant, were associated with higher affinity to NKG2D and classified as Type-I MICA, which increased the activating potential of the receptor [39]. A recent study showed the association between MICB G406A polymorphism and COVID-19 severity. It was observed that an increasing number of copies of MICB G406A variant alleles was connected with a lower risk of development of severe outcomes of SARS-CoV-2 infection [40].

Ligands for the NK cell receptors can be expressed as non-membrane-bound soluble molecules when they lack their transmembrane domains. In such soluble forms, they act as decoys for NK cell receptors, decreasing their potential to bind with target cells. Here, we observe the negative impact of high serum sMICB on SARS-CoV-2 infection, as the infected individuals had significantly increased serum sMICB concentration when compared to healthy controls (*p* = 0.0083). Elevated serum sMICB levels lead to impairment of the NKG2D activating receptor, blocking the binding of the receptor with its ligand; hence, high serum sMICB levels are connected with decreased cytotoxic potential of the NK cells. Indeed, we previously showed that serum sMICB levels were significantly higher in allogeneic hematopoietic stem cell transplantation recipients who were diagnosed with CMV infection and chronic graft-versus-host disease [41]. These observations indicate the negative impact of high serum sMICB concentration.

Although we did not observe any significant associations of sMICA or sHLA-E concentrations in the present study, other reports highlighted their importance in some clinical settings [42]. Soluble forms of other NKG2D-related ligands, ULBP2 and ULBP3, were also found to be significantly increased among COVID-19 patients suffering from severe infection, and their levels correlated with decreased expression of NKG2D [43].

This study has, however, some limitations that should be addressed. First, the relatively small sample size limits the statistical power of our analyses and increases the risk of limiting the detection of weaker associations between genetic variants and clinical outcomes. Second, the lack of comprehensive demographic and clinical variables (e.g., comorbidities and detailed medication records) also constrained our ability to adjust for potential confounders. As this was a single-centre study, our findings may not be fully generalizable to larger, more diverse patient populations; however, they point to a promising direction for further research, which may yield clinically valuable results. Future studies with larger sample sizes are needed to validate and expand upon our observations.

In conclusion, our study confirms previous observations regarding the impact of *NKG2C* and *HLA-E* genetic variability on the risk for SARS-CoV-2 infection, the development of COVID-19, and the severity of its symptoms. In addition, the effect of NKG2A and MICA genetic variants was observed. We also documented higher serum concentrations of sMICB in infected individuals. These results highlight the role of the NKG2 family of receptors and their ligands in SARS-CoV-2 infection.

## Figures and Tables

**Table 1:** Detailed SNP characteristics.

SNP ID	Gene	Nucleotide/Amino Acid Change	Localization
rs7301582	*NKG2A*/*KLRC1*	C>T	Intronic variant
rs1049174	*NKG2D*/*KLRK2*	C>G (LNK/HNK) ^	3′UTR
rs1264457	*HLA-E*	A>G/Arg107Gly (HLA-E*01:01/*01:03)	Exon 3
rs1051792	*MICA*	G>A/Val192Met	Exon 3
rs1065075	*MICB*	A>G/Lys48Glu	Exon 2

^ LNK—low NK cell activity; HNK—high NK cell activity.

**Table 2:** Detailed distributions of genetic variants.

SNP	Genotype	Asymptomatic	Home-Treated	Hospitalized	Healthy Controls
*NKG2A* rs7301582	*CC* *CT* *TT*	27 (72.97%) 8 (21.62%) 2 (5.41%)	40 (57.14%) 29 (41.43%) 1 (1.43%)	6 (60%) 4 (40%) 0 (0%)	56 (74.67%) 16 (21.33%) 3 (4%)
*NKG2C* deletion	wt/wt wt/del del/del	25 (67.57%) 12 (32.43%) 0 (0%)	44 (62.86%) 25 (35.71%) 1 (1.43%)	6 (60%) 4 (40%) 0 (0%)	58 (77.33%) 16 (21.33%) 1 (1.33%)
*NKG2D* rs1049174	*GG* *GC* *CC*	20 (54.05%) 14 (37.84%) 3 (8.11%)	31 (44.29%) 33 (47.14%) 6 (8.57%)	4 (40%) 6 (60%) 0 (0%)	36 (48%) 34 (45.33%) 5 (6.67%)
*HLA-E* rs1264457	*AA* *AG* *GG*	12 (32.43%) 17 (45.95%) 8 (21.62%)	34 (48.57%) 29 (41.43%) 7 (10%)	3 (30%) 6 (60%) 1 (10%)	21 (28%) 36 (48%) 18 (24%)
*MICA* rs1051792	*GG* *GA* *AA*	16 (43.24%) 15 (40.54%) 6 (16.22%)	29 (41.43%) 31 (44.29%) 10 (14.29%)	0 (0%) 9 (90%) 1 (10%)	26 (34.67%) 39 (52%) 10 (13.33%)
*MICB* rs1065075	*AA* *AG* *GG*	16 (43.24%) 17 (45.95%) 4 (10.81%)	33 (47.14%) 27 (38.57%) 10 (14.29%)	4 (40%) 3 (30%) 3 (30%)	29 (38.67%) 39 (52%) 7 (9.33%)

**Table 3:** Serum concentrations of soluble forms of the HLA-E, MICA, and MICB ligands.

		Mean [pg/mL]	SD	Median [pg/mL]
**sHLA-E**	non-infected	56.19	±16.68	54.39
	asymptomatic	60.54	±16.53	64.01
	home-treated	56.64	±15.73	55.10
	hospitalized	53.83	±28.05	62.41
**sMICA**	non-infected	47.85	±21.45	45.78
	asymptomatic	66.13	±33.57	71.42
	home-treated	48.63	±36.90	33.96
	hospitalized	42.52	±53.00	20.68
**sMICB**	non-infected	86.74	±14.50	82.28
	asymptomatic	114.8	±52.99	99.35
	home-treated	116.2	±34.55	112.5
	hospitalized	90.67	±9.083	88.53

## Data Availability

Data available on request from the authors.
